# Effect of Vitamin K_3_ Inhibiting the Function of NorA Efflux Pump and Its Gene Expression on *Staphylococcus aureus*

**DOI:** 10.3390/membranes10060130

**Published:** 2020-06-25

**Authors:** Saulo R. Tintino, Veruska C. A. de Souza, Julia M. A. da Silva, Cícera Datiane de M. Oliveira-Tintino, Pedro S. Pereira, Tereza C. Leal-Balbino, Antonio Pereira-Neves, José P. Siqueira-Junior, José G. M. da Costa, Fabíola F. G. Rodrigues, Irwin R. A. Menezes, Gabriel C. A. da Hora, Maria C. P. Lima, Henrique D. M. Coutinho, Valdir Q. Balbino

**Affiliations:** 1 Laboratory of Microbiology and Molecular Biology (LMBM), Department of Biological Chemistry/CCBS/URCA, Crato 63105-000, Brazil; saulorelison@gmail.com (S.R.T.); datianemorais@hotmail.com (C.D.d.M.O.-T.); pedro.sillvino@gmail.com (P.S.P.); irwinalencar@yahoo.com.br (I.R.A.M.); 2 Fiocruz, Departamento de microbiologia, Instituto Aggeu Magalhães , Recife 50740-465, Brazil; veruska.cintia02@hotmail.com (V.C.A.d.S.); jmariana.assis@gmail.com (J.M.A.d.S.); tcristinaleal@gmail.com (T.C.L.-B.); antoniopnn@yahoo.com.br (A.P.-N.); 3 Laboratory of Microrganism Genetics (LGM), Department of Molecular Biology/CCEN/UFPB; João Pessoa 58051-900, Brazil; siqueira@dbm.ufpb.br; 4 Laboratory of Natural Products (LPPN), Department of Biological Chemistry/CCBS/URCA, Crato 63105-000, Brazil; galberto.martins@gmail.com (J.G.M.d.C.); fabiola@leaosampaio.edu.br (F.F.G.R.); 5 Department of Chemistry, University of Utah, Salt Lake City, UT 84112, USA; gabriel.costa.hora@gmail.com; 6 Department of Medicinal Chemistry, University of Utah, Salt Lake City, UT 84112, USA; mcpl13@gmail.com; 7 Department of Genetics, Federal University of Pernambuco, Recife 50670-901, Brazil; vqbalbino@gmail.com

**Keywords:** efflux pump, ethidium bromide, menadione, NorA, *S. aureus*

## Abstract

Resistance to antibiotics has made diseases that previously healed easily become more difficult to treat. *Staphylococcus aureus* is an important cause of hospital-acquired infections and multi-drug resistant. NorA efflux pump, present in bacteria *S. aureus*, is synthesized by the expression of the *norA* gene. Menadione, also known as vitamin K_3_, is one of the synthetic forms of vitamin K. Therefore, the aim of this study is to verify the menadione effect on efflux inhibition through NorA pump gene expression inhibition and assess the effects of menadione in bacterial membrane. The effect of menadione as an efflux pump inhibitor (EPI) was evaluated by the microdilution method, fluorimetry, electron microscopy, and by RT-qPCR to evaluate gene expression. In the molecular docking, association with menadione induces increased fluorescence intensity. Menadione was observed (100% of the clusters) interacting with residues ILE12, ILE15, PHE16, ILE19, PHE47, GLN51, ALA105, and MET109 from NorA. The results showed the *norA* gene had its expression significantly diminished in the presence of menadione. The simulation showed that several menadione molecules were able to go through the bilayer and allow the entry of water molecules into the hydrophobic regions of the bilayer. When present within membranes, menadione may have caused membrane structural changes resulting in a decline of the signaling pathways involved in *norA* expression. Menadione demonstrated to be an efflux pump inhibitor with dual mechanism: affecting the efflux pump by direct interaction with protein NorA and indirectly inhibiting the *norA* gene expression, possibly by affecting regulators present in the membrane altered by menadione.

## 1. Introduction

Antibiotics used for several years were effective against countless infectious diseases caused by several different microorganisms, such as *Staphylococcus aureus* [1]. *Staphylococcus aureus* is an important cause of community bacterial infections and is also related to infections in hospitals [2]. The infections caused by methicillin-resistant *S. aureus* (MRSA) affect millions of people worldwide, provoking serious problems in the skin, bones, lungs, and soft tissue [3]. The fluoroquinolone class of antimicrobial agents were, for many years, an effective option for therapy against *Staphylococcus aureus*. [4]. However, after introduction of these agents into clinical large-scale use, the emergence of fluoroquinolone-resistant *S. aureus* was observed [5].

Therefore, the emergence of antibiotic resistance made some diseases that were previously treated into a major public health problem. One of the main mechanisms related to antibiotic resistance involves membrane proteins that pump drugs from the bacterial cell interior out of the cell. Known as efflux pumps, they are transmembrane proteins involved mainly involved in the extrusion of toxic compounds, and certainly about acts all classes of antibiotics [6]. It is important to mention that the efflux pump has physiological functions in bacteria and includes the secretion of virulence factors in cell stress responses. Therefore, it is believed that the drugs are “accidental substrates” of these transporters. These proteins can be found in Gram-positive and Gram-negative bacteria. In addition, they are found in eukaryotic cells [7].

Efflux pumps can exist in bacteria through acquire resistance at two levels: intrinsic and acquired. The intrinsic resistance is the natural resistance of the bacteria acquired in a vertical manner, through from its primitive cell along the evolution of population [8]. The acquired bacterial resistance involves increased levels of resistance, and can be achieved by the acquisition; therefore, through horizontal gene transfer (for example, as the gene for a new efflux pump, which, acquired by a given strain, through plasmid). *Staphylococcus aureus* presents the ability to acquire efflux pumps through both forms mentioned above [6]. The NorA efflux pump, present in bacteria *S. aureus*, is synthesized by the expression of the *norA* gene, located in the Small D fragment of the *S. aureus* chromosome, is a member of the large facilitating superfamily (MFS). It is known to play an important role in the development of quinolone resistance by reducing its concentration within the target pathogen [9].

In addition to research efforts towards the development of new antibiotics, there is also interest in discovering efflux pump inhibitors (EPIs). Similar to the b-lactamase combinations currently in clinical use, EPIs could be combined with existing drugs to restore antibiotic susceptibility to efflux-proficient strains [10]. Such combination treatment could reduce the emergence of antibiotic-resistant strains, broaden antibacterial spectra, and reduce biofilm formation and resistance. Efflux pump inhibition can be achieved by reducing pump gene expression, interrupting pump assembly, reducing substrate binding by competitive or non-competitive means, and disrupt the pump’s required energy source [11]. It is also possible that some EPIs have affinity for efflux pump substrates, and that they may form EPI–substrate complexes that prevent substrate extrusion from the cell [12].

Menadione, also known as vitamin K_3_, is one of synthetic forms of vitamin K. The menadione has, as a main characteristic, to be a fat-soluble vitamin. It has quite long been recognized for its essential function in coagulation and, moreover, has been pointed at as an involved nutrient in the control of tissue calcification [13]. Vitamin K is a nutrient considered safe for human consumption only at recommended adequate doses. The upper limit set is not known because there are no known cases of clinical toxicity with menadione. With great potential for use, the menadione is available in formulations such as: capsules, topical creams, softgels, and tablets [13]. It is known in the literature that lipophilic substances cause changes in the membranes of prokaryotes, and it is also known that membrane proteins, such as efflux proteins, are sensitive to membrane changes [14].

Therefore, the objective of the present study is to verify the menadione effect on efflux inhibition through NorA pump gene expression inhibition, and to show how its action on the cell membrane is associated with the inhibition of this pump using transmission electron microscopy.

## 2. Materials and Methods

### 2.1. Bacterial Strain and Culture

The SA-1199B strain of *Staphylococcus aureus* was kindly donated by Prof. S. Gibbons of the University of London. Before all microbiological tests, the strain was maintained in the culture medium blood agar base (Laboratorios Difco Ltd., São Paulo, Brazil). For use in testing, the cells were grown overnight at 37 °C in the culture medium: Heart Infusion Agar (HIA, Difco). The strain used has the generic characteristic of presenting the *norA* resistance gene responsible for expressing the efflux pump (NorA) for antibiotics in the group of fluoroquinolones.

### 2.2. Drugs

The menadione and antibiotic was purchased through the manufacturer Sigma Aldrich Co. Ltd (St. Louis, MO, USA). The norfloxacin and menadione were dissolved in the amphipathic solvent dimethyl sulfoxide (DMSO) with an initial concentration of 10,000 μg/mL. Norfloxacin was the antibiotic chosen because it is a substrate of the pump. These solutions were subsequently diluted in sterile (distilled) water until they reached the concentration of 1024 μg/mL, and posteriorly stored at −20 °C until the time of testing.

### 2.3. Evaluation of Efflux Pump Inhibition by Minimum Inhibitory Concentration (MIC) Reduction of Ethidium Bromide (EtBr)

To determine whether menadione is a potential efflux pump inhibitor, the inhibition of the efflux pump was tested by updating a sub-inhibitory concentration (MIC/8). The MIC/8 value used was according to the study by Tintino et al. [15]. The distribution medium was prepared using a solution of bacterial inoculum in saline solution, in a concentration of 1.5 × 10^8^, according to the McFarland scale. The preparation of the eppendorfs was followed by the addition of 150 μL of the inoculum and 1350 μL of the culture medium, brain-to-brain infusion (BHI), this being the control. In the preparation of the test eppendorfs, in addition to the inoculum and culture medium, menadione was added at a subinhibitory concentration (MIC/8) totaling a volume of 1500 μL. Then, 100 μL of eppendorf content was distributed in titration microplates, distributed in triplicate, followed by serial microdilution with EtBr (1:1). Finally, the plates were incubated in an oven for 24 h at 37 °C and read with the addition of resazurin. The significant MIC was considered as the lowest concentration between 0.5 and 512 μL of EtBr capable of inhibiting bacterial growth.

### 2.4. Statistical Analysis of Evaluation of Efflux Pump Inhibition by MIC Reduction of Ethidium Bromide

The geometric mean of the results was used. The statistical analysis was carried out through the GraphPad Prism 5.0 software, applying two-way ANOVA, and the post hoc Bonferroni.

### 2.5. Evaluation of Pump Inhibition by Fluorescence Emission of Ethidium Bromide

Fluorescence spectra were obtained on a Shimadzu RF6000 spectrofluorometer with a 150 w xenon lamp for sample excitation. The samples were packed in a quartz cuvette (10 mm optical path) of four polished faces and subjected to scattered excitation light with a maximum wavelength of 530 nm, scanning speed of 600 nm/min and spectral resolution 3 nm with an increase of 0.5 nm. The emission was monitored from 400 to 800 nm. Fluorescence evaluation preparations were cultured in Brain Heart Infusion (BHI) culture medium for 24 h at 37 °C and were subsequently centrifuged at 3000 rpm (Fanem Centrífuga Excelsa^®^ 3280, Carandiru, São Paulo - SP, Brazil) at 3 min for pellet formation and resuspended in saline. The substances prepared were: 1—microorganism with ethidium bromide in saline solution; 2—microorganism associated with ethidium bromide in (MIC/8) and menadione in (MIC/8) in saline solution.

### 2.6. Homology Modeling and Molecular Docking

The interaction of NorA efflux pump with menadione was investigated at atomistic level using Molecular Docking. Due to the unavailability of crystal structure of *S. aureus* NorA, the three-dimensional model was constructed by homology modeling from the YajR transporter structure (PDB ID: 3WDO on the Protein Data Bank) [16]. The structure was modeled using the SWISS-MODEL (bioinformatics web server) protein structure homology-modelling server [17,18]. The optimized structure of the menadione was obtained from Automated Topology Builder (ATB) Repository version 3.0 [19]. The molecular docking calculation was performed using the AutoDock 4.2 [20,21] and AutoGrid4 [21] combined with the AutoDock Tools package [20,22]. The dihedral torsions were treated as completely flexible for menadione. The partial charges of the ligand were calculated using the Gasteiger method [23], while the NorA atom charges were assigned according to the AMBER86 force field parameters [24]. The ligand was positioned in the center of the channel formed by the receptor helices at −28,335/56,567/73,952 Å and used to obtain the affinity maps (grid maps of 126 × 126 × 126 points and point spacing of 1.42 Å). In order to obtain the docked conformation, 100 runs were performed using the Lamarckian genetic algorithm. The parameters used on those runs were: initial population of 150 random individuals, 2,500,000 energy evaluations (maximum), 27,000 generations with mutation rate of 0.02, and crossover rate of 0.08. The probability of performing a local search on an individual was 6%. The maximum number of consecutive successes or failures before halving or doubling the search step was 4. While the maximum iterations allowed per local search was set to 300. A root-mean-square-deviation tolerance of 2.0 Å was used to cluster the coordinates of the lowest energy conformation [20,25].

### 2.7. Sample Preparation for the Real-Time PCR Assay

The samples were prepared using the minimum inhibitory concentration (MIC) as obtained in Tintino et al. [15]. The assays were performed as described in the Section 2.5. The preparations were cultured in Brain Heart Infusion (BHI) culture medium for 24 h at 37 °C and then centrifuged at 3000 rpm (Fanem Centrífuga Excelsa^®^ 3280) for pellet formation, procedures performed prior to RNA extraction.

### 2.8. Gene Expression Evaluation

RNA extraction from the *S. aureus* strain was performed using the SV Total RNA Isolation System (Promega, Madison, WI, USA) based to the manufacturer’s indications. Complementary DNA (cDNA) was synthesized from total RNA by reverse transcription (RT) using the GoScript called Transcription System (Promega, Madison, WI, USA). NorA expression was assessed by qPCR, performed in triplicates using a Power SYBR^®^ Green PCR Master Mix (Applied Biosystems Co., Foster City, CA, USA) and an ABI PRISM 7500 sequence detector (Applied Biosystems, Foster City, CA, USA). The amount of cDNA was used as a template in 20 μL reaction, along with 10 μL Power SYBR^®^ Green and 1 pmol of each primer. The *16S* gene was chosen to be used as an endogenous control. Table 1 shows the primers used in this study. The relative *norA* gene expression was determined using the ΔΔTc method.

### 2.9. Transmission Electron Microscopy

The samples were prepared using the minimum inhibitory concentration (MIC) as obtained in Tintino et al. [15]. The assay was performed using: (a) cells grown in culture medium under standard conditions; (b) cells treated with a subinhibitory (MIC/4) concentration of the antibiotic of 16 μg/mL; (c) cells treaded with 2 μg/mL menadione for (MIC/4); (d) cells treated with a subinhibitory (MIC/4) concentration of the antibiotic associated with menadione at a subinhibitory (MIC/4) concentration; and a growth control, the micro-organism alone. The preparations were cultured in Brain Heart Infusion (BHI) culture medium for 24 h at 37 °C and were subsequently centrifuged at 3000 rpm (Fanem Centrífuga Excelsa^®^ 3280) for pellet formation.

Cells were washed with PBS buffer and subsequently fixed in the concentration of 2.5% glutaraldehyde, 4% paraformaldehyde in 0.1 M cacodylate buffer, with pH 7.2, overnight. The cells were then fixed for 30 min in the concentration of 1% OsO4, with dehydration in acetone, and embedded in solution of Polybed 812. In the following step, ultra-thin sections were selected on 300-mesh copper grids, and stained with uranyl acetate (concentration of 5%) and lead citrate (concentration of 1%). Posteriorly were observed with an FEI Tecnai G2 Spirit transmission electron microscope, operating at 120 kV. The images were shape chosen at random through a CCD camera system (MegaView G2, Olympus, Tokyo, Japan).

### 2.10. Molecular Dynamics Simulation

An atomistic simulation of Molecular Dynamics (MD) was performed to investigate the effect of menadione on bacteria cell membranes. The simulation box consisted of 128 menadione units arranged approximately 4 Å apart from a previously equilibrated bilayer of 512 1-palmitoyl-2-oleoyl-sn-glycero-3-(phospho-rac-(1-glycerol)) (POPG) units [28]. The atomic parameters of menadione were obtained in ATB, while the Kukol model [29] was used for the phospholipids. The simulation was performed for 400 ns using the GROMOS 54A7 force field parameters set [30] to describe the bonded and non-bonded interactions. The system was hydrated with the simple point-charge (SPC) water model and was neutralized with 512 Na^+^ counter ions described by the GROMOS 53A6 parameter set [31]. The system initially had its energy minimized by 10,000 steps and then simulated with periodic boundary conditions applied in all directions. During the equilibration and production phases, a time step of 2 fs was applied to integrate the equations of motion under NPT conditions, i.e., keeping the number of particles (N), pressure (P) and temperature (T) constant. The temperature was maintained constant at 298 K through the canonical velocity-rescaling thermostat [32], coupling separately the temperatures of each component (ligand, protein, water, and ions) with a time constant of 0.2 ps for each. The Berendsen barostat [33] was used to maintain constant pressure at 1.0 bar through the semi-isotropic coupling (*xy* plane independent of *z*) at every 0.1 ps. A relaxation time was 0.1 ps and a compressibility of 4.5 × 10^−5^ (kJ·mol^−1^·nm^−3^)^−1^ was applied, as appropriate for water [33]. The long-range interactions and van de Waals were treated with Verlet cutoff scheme (cutoff = 1.0 nm). The Particle-Mesh Ewald [34] was applied to treat long-range electrostatic interactions with a dielectric constant of 66 [35].

The simulation and all the analyses (partial density profiles, deuterium order parameter and tilt angle of lipids) were performed using the GROMACS 5.1.5 software, [36] except the tilt angle which was used the script developed by Pontes [37]. The software VMD, version 1.9.1 [38] was used to visualize the coordinates, trajectories and for the screenshots.

## 3. Results

### 3.1. Efflux Pump Inhibition by MIC Reduction of Ethidium Bromide (EtBr)

**Figure 1:**
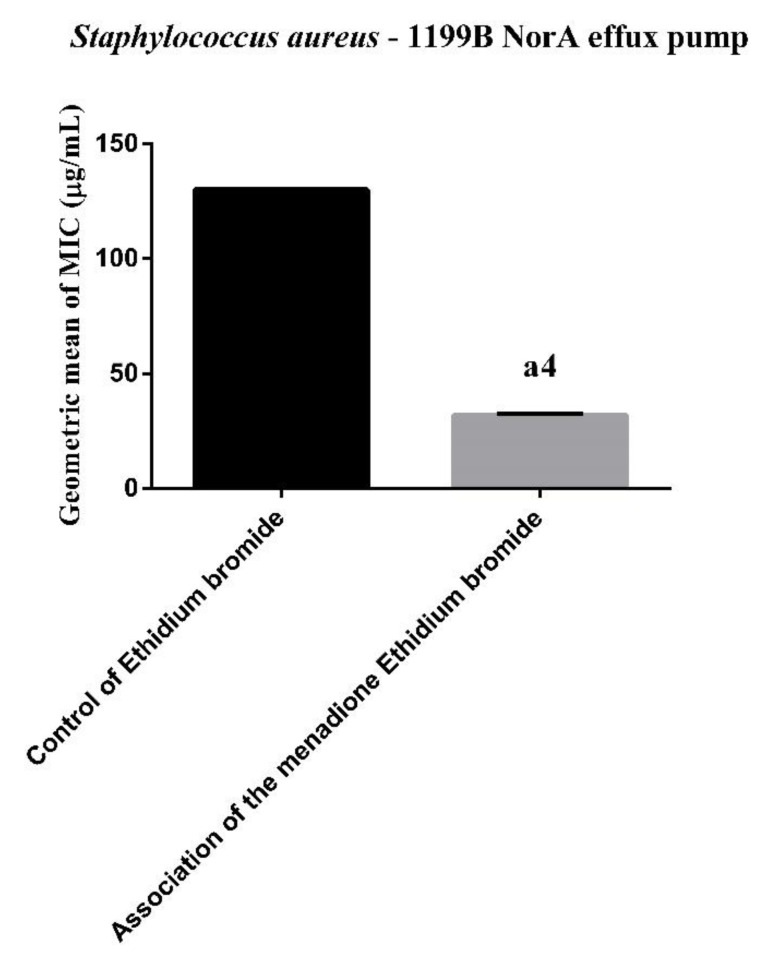
Synergistic effect of menadione on the ethidium bromide against the strain of *S. aureus* 1199B. Geometric mean ± standard error of the mean, with *t*-Test. a4: *p* < 0.0001 vs. control of ethidium bromide.

In the evaluation of inhibition by MIC reduction of ethidium bromide, the results in Figure 1, menadione was shown to significantly reduce the ethidium bromide MIC, which indicates NorA inhibition. In the inhibition of the efflux pump, it is known that decreasing the MIC of bromide, of ethidium or antibiotic, by up to three-fold, in relation to the control (substrate of efflux pump) alone, is indicative of efflux pump inhibition.

### 3.2. Evaluation of Pump Inhibition by Fluorescence Emission of Ethidium Bromide

**Figure 2:**
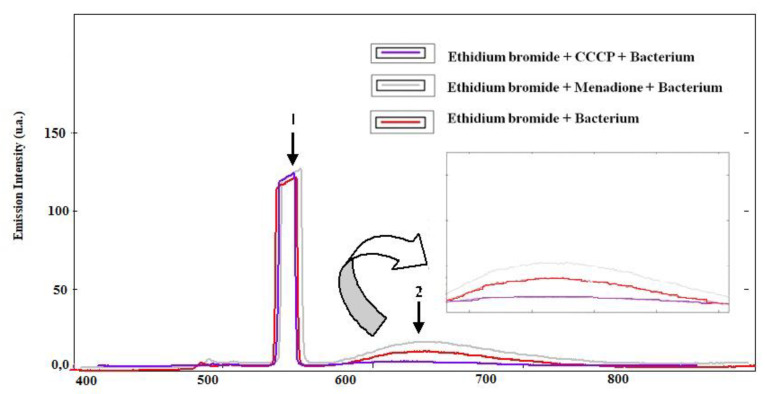
Inhibition of efflux pump by increasing fluorescence. 1—Peak corresponding to the other bacterial constituents. 2—Peak corresponding to ethidium bromide. Inhibition of efflux pump by increasing fluorescence. Carbonyl Cyanide m-Chlorophenylhydrazine (CCCP).

In the evaluation of inhibition by fluorescence emission of ethidium bromide, according to the results in Figure 2, the association with menadione induces increased fluorescence intensity when compared to the bacterium associated only with bromide, indicating inhibition of efflux pump. Regarding the Carbonyl Cyanide m-Chlorophenylhydrazine (CCCP) pump inhibitor, the same showed low fluorescence intensity when compared to the others.

### 3.3. Homology Modeling and Molecular Docking

**Figure 3:**
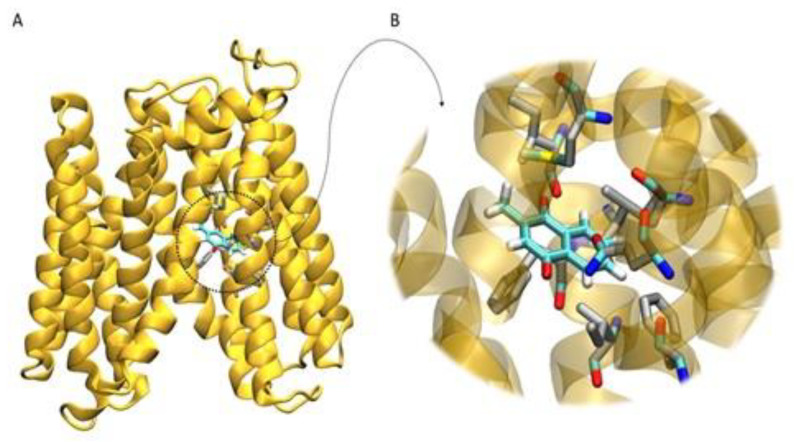
Molecular docking of the structural model for the complex menadione-nora. (**A**) Overall structure of NorA obtained through homology modeling, with the position of Menadione inside of the transmembrane channel. (**B**) Detail of the lowest energy conformation and most populated conformational cluster obtained from the molecular docking calculations. Menadione is interacting with the closer residues ILE12, ILE15, PHE16, ILE19, PHE47, GLN51, ALA105, and MET109. Ligand is represented in cyan. Receptor molecule is represented in yellow, while the residues closest to the ligand are represented in gray. Hydrogen, oxygen, nitrogen and sulfur are represented in white, red, blue and yellow, respectively.

The molecular docking method was used to investigate the binding affinity of menadione in the channel formed by the transmembrane alpha-helices of the NorA protein. Menadione was observed (100% of the clusters) interacting with residues ILE12, ILE15, PHE16, ILE19, PHE47, GLN51, ALA105, and MET109 from NorA (Figure 3). This protein region has also been observed in other docking studies in the literature with other binders such as boeravinone B [39], riparin B [40], salvin, thioxanthene, and levofloxacin [41].

### 3.4. NorA Quantitative Real-Time PCR

**Figure 4:**
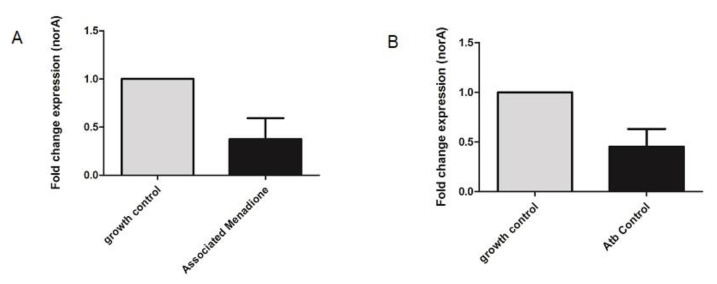
Relative *norA* gene expression in *S. aureus* 1199B in the presence of menadione associated with norfloxacin in comparison to the growth control: (**A**) comparison between growth control and association; (**B**) comparison between growth control and norfloxacin alone.

The relative *norA* gene expression in *S. aureus* strains was evaluated by RT-qPCR analysis in the presence of menadione. The results showed the *norA* gene had its expression significantly diminished in the presence of menadione (Figure 4/Table 2). In the presence of the other compounds, a reduction in *norA* gene expression was observed; however, this was not statistically significant.

### 3.5. Transmission Electronic Microscopy

**Figure 5:**
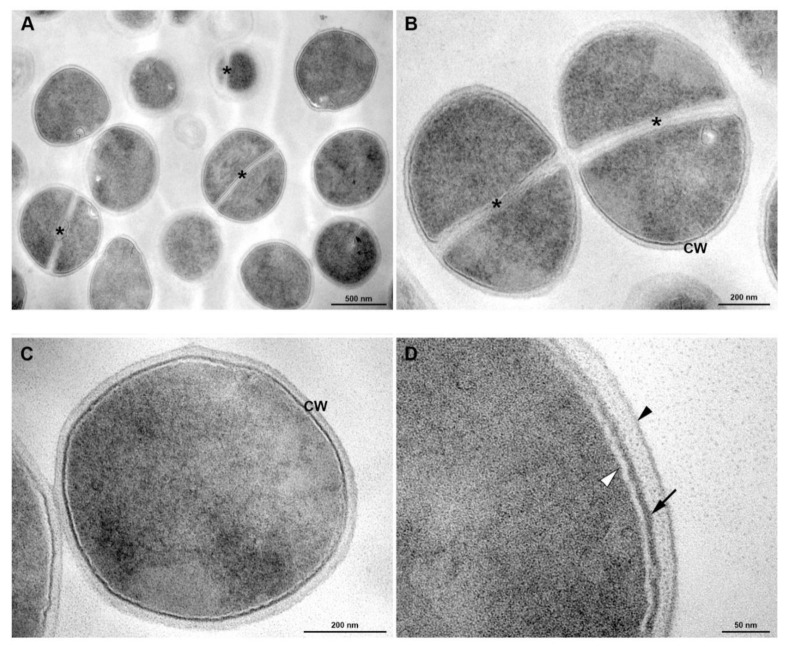
Transmission electron microscopy of *S. aureus* grown under culture medium. (**A**) General view showing rounded cells with a thick cell wall envelope and homogeneous electron density in the cytoplasm. Central division septa (*) are seen in some cells. (**B**,**C**) Detailed view of non-dividing (**B**) and dividing (**C**) bacteria A tripartite cell wall (CW) is seen enclosing the plasma membrane. Asterisk indicates the septum. (**D**) Inset of the cell wall. Black arrowhead indicates outer highly stained fibrous surface and intermediate translucent region; Arrow points to a heavily stained inner thin zone; the plasma membrane (white arrowhead) is seen immediately below this electrodense layer of the wall. Bars: A: 500 nm; B,C: 200 nm; D: 100 nm.

**Figure 6:**
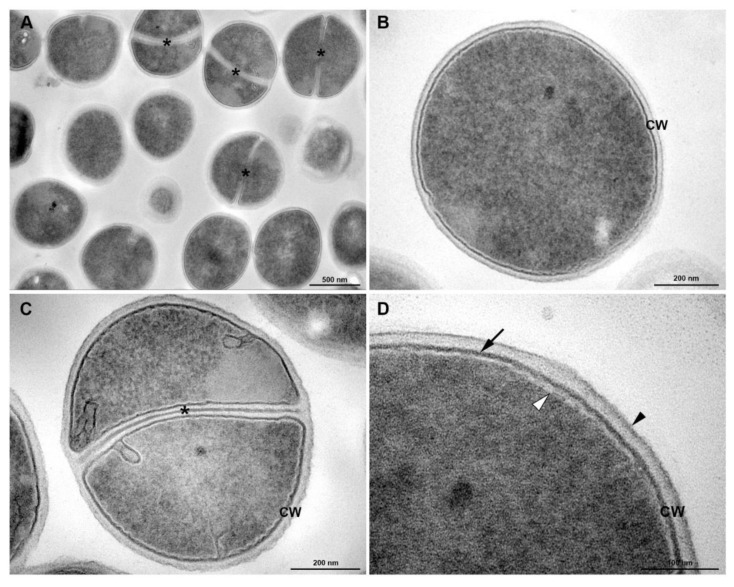
Transmission electron microscopy of *S. aureus* treated with antibiotics (Norfloxacin MIC/4): (**A**) General view: the cells are round and intact, with a well-defined cell wall. Division septa (*) are seen in some cells. (**B**,**C**) Detailed view of dividing (**B**) and non-dividing (**C**) bacteria showing the tripartite cell wall (CW). Asterisks indicate cell wall septa. (**D**) Inset of the cell wall. Black arrowhead, outer highly stained fibrous surface and intermediate translucent region; Arrow, heavily stained inner thin zone; white arrowhead, plasma membrane. Bars: A: 500 nm; B,C: 200 nm; D: 100 nm.

In the evaluation of membrane damage, transmission electronic microscopy showed in Figure 5; homogeneous cell walls without any evident alterations and with intact cytoplasm can be observed in the control. Untreated cells of *S. aureus* grown under culture medium displayed the typical features of *Staphylococci* morphology: rounded cells with an intact thick cell wall envelope and well-defined membranes (Figure 5C,D). The cytosol exhibited a homogeneous electron density (Figure 5C,D) and proliferating cells with a central septum were commonly observed (Figure 5A,B). The cell wall showed a tripartite structure consisting of an outer that shows highly stained fibrous surface, an intermediate translucent region and an electrodense inner thin zone (Figure 5B–D). The last region was more stained, and the visualization of the plasma membrane was difficult. The plasma membrane was observed immediately below this electrodense layer of the wall (Figure 5D). Similar results were found in *S. aureus* treated with norfloxacin (Figure 6).

**Figure 7:**
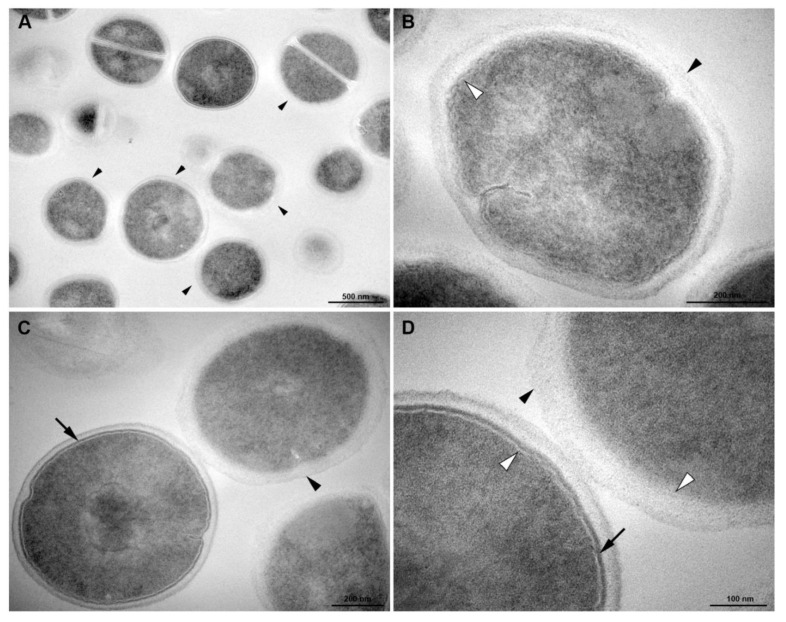
Transmission electron microscopy of *S. aureus* treated with antibiotics (Menadione MIC/4). (**A**) General view: some cells display altered cell wall without a tripartite-layers structure (black arrowheads). (**B**) Detailed view: the cell has no visible electron-dense inner thin zone of the cell wall (black arrowhead). White arrowhead indicates the plasma membrane. (**C**) Left cell display the normal tripartite cell wall with a heavily stained inner thin zone (arrow), whereas this electron-dense layer is not seen on the right cell (black arrowhead). (**D**) Inset of the Figure C. White arrowhead, plasma membrane. Bars: A: 500 nm; B,C: 200 nm; D: 100 nm.

After menadione treatment, *S. aureus* remained intact, exhibiting rounded morphology and homogeneous electron density in the cytosol (Figure 7). However, some cells showed alterations in the cell wall: without a tripartite-layers structure and no visible electrodense inner thin zone (Figure 7). In these cells the visualization of the plasma membrane was easier than those under control conditions (Figure 7B). Changes in the outer highly stained fibrous surface and intermediate translucent region of the cell wall were also observed (Figure 7C,D).

**Figure 8:**
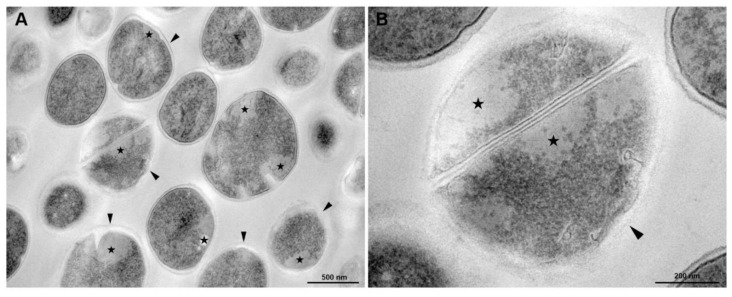

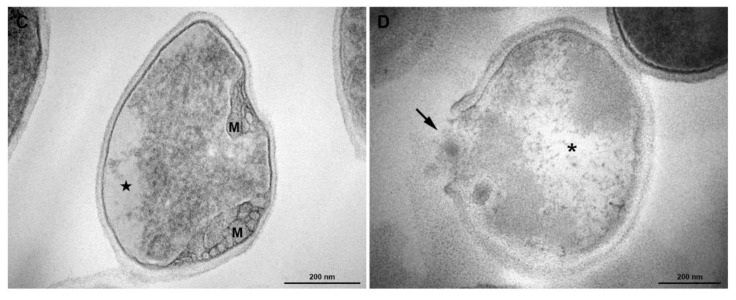
Transmission electron microscopy of *S. aureus* treated with antibiotics (Menadione MIC/4) and Antibiotic CIM/4). (**A**) General view: some cells exhibit abnormal electron density in the cytoplasm (★) and altered cell wall with no visible tripartite-layers structure (black arrowheads). (**B**) Inset of Figure A. (**C**) Detailed view of a cell showing alterations in the shape, loss of cytosolic electron-density (★) and mesosome-like structures (M). (**D**) A lysed cell with cell wall disruption (arrow) and cytoplasmic disintegration (asterisk). Bars: A: 500 nm; B–D: 200 nm.

According to Figure 8, menadione treatment in association with antibiotic provoked severe morphological alterations in *S. aureus*. The cells showed abnormal electron density in the cytoplasm and cell wall with no visible tripartite-layers structure (Figure 8A,B). Changes in the shape and mesosome-like structures were also observed (Figure 8C). Cytoplasm disintegration and lysed cells were often seen (Figure 8D).

### 3.6. Molecular Dynamics Simulation

**Figure 9:**
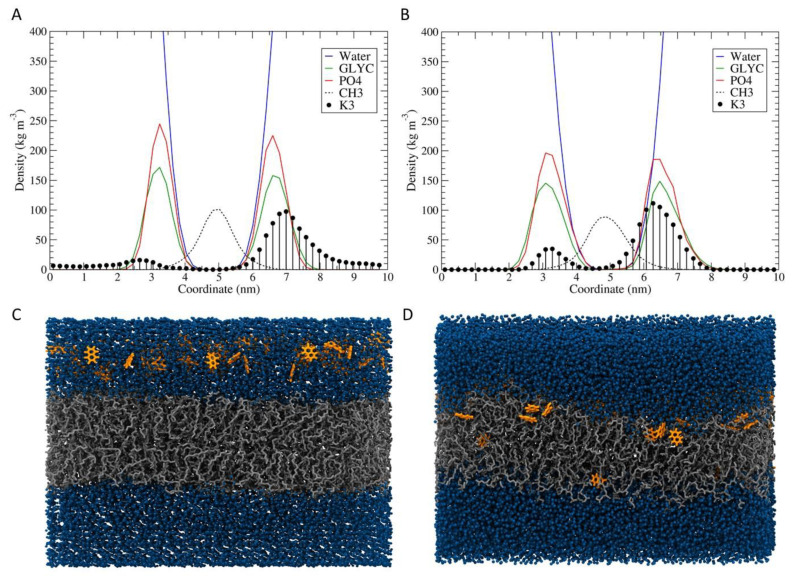
Average density profiles and snapshots conformations for the beginning and the end of menadione with POPG bilayer simulation. Initially (**A**,**C**) The menadione molecules (orange) were distributed above the membrane (grey). In the end (**B**,**D**), several went through the membrane and allowed the entry of water molecules (blue spheres) in the hydrophobic regions. The density curves shown are: water (blue lines), phosphate groups (red), glycerol groups (green), acyl chains (black dots), and menadione molecules (black circles).

In order to explore the results obtained by the electron transmission microscopy, MD simulation of menadione interacting with phospholipids membrane was performed. The simulation showed that several menadione molecules were able to go through the bilayer and allow the entry of water molecules into the hydrophobic regions of the bilayer (Figure 9).

## 4. Discussion

Ethidium bromide is an efflux pump substrate which, when present intracellularly, is extruded by efflux pumps allowing bacterial cell survival, even with high bromide concentrations in the medium [42]. This fact was verified in the present study. A similar MIC reduction result was observed in the study by Tintino et al. [15], however, in the present study this reduction was even greater.

The verification of pump inhibition by increased fluorescence is already widespread in the literature. It is known that the ethidium bromide when inside the cell can intercalate with the bacterial DNA. Binding to DNA induces increased fluorescence of ethidium bromide [43]. Efflux pumps remove ethidium bromide and other toxic substances from the bacterial interior. Pump inhibition results in a greater amount of bromide in the interior, making a larger number of molecules attachable to DNA; this results in a higher fluorescence [44].

The affinity for this area of the protein can be explained by the hydrophobic character of the menadione and the region, with the presence of aliphatic residues (several isoleucine, alanine) and an aromatic (phenylalanine). In addition, this feature allows the menadione to interact with other menadione through π-π interactions of its aromatic rings within the efflux pump. Therefore, this result indicates that this interaction of menadione with NorA promotes the pump inhibition, observed through MIC reduction and fluorescence emission of ethidium bromide.

A decrease in gene expression is one of the growing strategies used for efflux pump inhibition. This may involve both direct efflux pump genetic synthesis inhibition, as well as by inhibition of the mediators necessary for efflux pump gene expression, i.e., interfering with the steps involved in gene regulation [45].

It is known today that efflux pump genes are regulated through more than one regulator, as is the case of the NorA gene, which is regulated mainly by the ArlR-A1rS two-component membrane sensor. Induction of *norA* gene expression by this sensor is still poorly understood, however it is believed to be mediated by an 18-kDa pathway protein. This protein binds to DNA sequences that are located upstream of the-35 region of the *norA* promoter [46].

It is believed that menadione may have acted indirectly on the gene expression inhibition. Menadione action has probably occurred in the membranes since it is a hydrophobic molecule which can therefore easily cross the bacterial plasma membrane [47]. When present within membranes, menadione may have caused membrane structural changes resulting in a decline of the signaling pathways involved in NorA expression since one of the major *norA* gene expression signals involves the ArlR-A1rS membrane sensor [48]. The membrane structural changes can be seen in Figure 7 and Figure 8 of the scanning electron microscopy.

Although norfloxacin can inhibit bacterial DNA replication and transcription by inhibiting topoisomerase II (a bacterial DNA-gyrase) [49], our results indicate that the antibiotic did not affect the viability, proliferation and cell structure of bacteria. This could be explained by the fact that *S. aureus* was treated with a subinhibitory concentration of norfloxacin. In addition, efflux pumps could be a major contributor in development of resistance against norfloxacin.

Menadione activity over *Staphylococcus aureus* has been previously mentioned, according to the study by Tintino et al. [15]. In the study, the author showed menadione activity with a value lower than 1024 μg/mL, this being, therefore, clinically relevant according to the author. In the study by Andrade et al. [50], menadione activity is shown against *S. aureus*, as well as against other Gram-positive and Gram-negative bacteria. As in the aforementioned study, the concentration mentioned herein is a low toxicity concentration, being well below the LC_50_ (Lethal concentration) obtained in vivo [51]. Therefore, this shows one of the advantages of using menadione, this being that its present low toxicity in the concentration used.

It is known in the literature that fat-soluble substances can induce changes to the membranes, resulting in change in membrane morphophysiology and cause damage, such as: reduced membrane potential as well as ion; cytochrome C; protein and radicals loss, and subsequently collapse of the proton pump with ATP depletion [14,52,53]. Therefore, it is possible that menadione acted directly on the membrane, according to the microscopy images

It is also important to emphasize that menadione caused other toxic effects to the bacterium, in addition to those previously mentioned, as observed through changes in the cytoplasm. In carcinogenic eukaryotic cells, menadione has a toxic effect through the production of reactive oxygen species. It is possible that this effect also occurred in the bacterial cell, which may justify part of the observed cytoplasmic damages [54].

In the study by Andrade et al. [50], menadione led to an increase in antibiotic activity by a membrane permeability mechanism. The author also indicated the use of menadione at different subinhibitory concentrations reverted bacterial resistance to different antibiotics, making the bacterium more sensitive to the antibiotic. Liposoluble compounds can cause degradation of the cell wall and lead to cellular content leakage, cytoplasm coagulation, and decrease in proton motive force. The NorA protein is part of the Major Facilitator Superfamily (MFS), and your source of energy is proton force H^+^ [55]. Therefore, the success in antibacterial potentiation by menadione with respect to multi-resistant *Staphylococcus aureus* strains may be explained, possibly by the blocking of the MFS efflux pump proton source, which causes inhibition of the pump in the SA-1199B strain, as well as destabilization of the cytoplasmic membrane.

**Figure 10:**
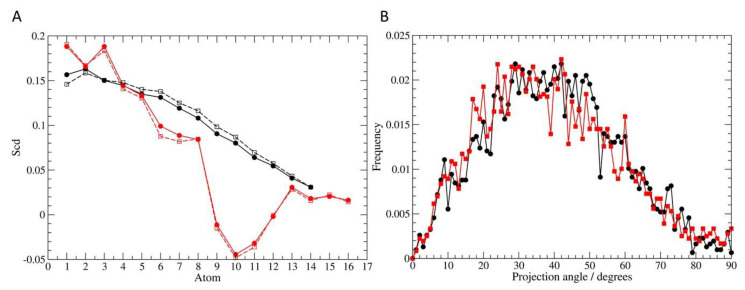
Degree of lipid reorientation and overall disorder in the bilayer. (**A**) Deuterium order parameter S_CD_ values were calculated for acyl chains sn1 (black) and sn2 (red). The filled circles and squares represents the order at the first 10 ns of simulation, while the empty circles and squares are the values for the last 10 ns; (**B**) Frequency distribution of the tilt angle of the lipid with the respect to the normal axis to the bilayer. The orientation values (0° = parallel to the *z* axis; 90° = perpendicular to the *z* axis) were also averaged for the initial 10 ns (black) and the final 10 ns (red).

At the beginning of the simulation (Figure 9A,C), the menadione molecules were arranged above the surface of the membrane and did not interact with the lipids carbon chains. Due to their hydrophobic character, the K_3_ molecules approached the membrane and began to access the aliphatic regions. Throughout the simulation, several molecules were observed crossing the bilayer, allowing the entry of water molecules in this process. Towards the end of the simulation, it was possible to verify the increase of the amount of menadione on the other side of the bilayer (Figure 9B,D). In addition, through the analysis of the deuterium order parameter and the tilt angle, it was found that menadione did not promote major perturbations in the acyl chains of the phospholipids (Figure 10). The results indicate that this process of crossing through membrane facilitates the exchange of content from external to internal environment. This exchange possibly promotes the destabilization of the cell membrane, as observed in the analysis by transmission electron microscopy.

## 5. Conclusions

The menadione may have caused membrane structural changes resulting in a decline of the signaling pathways involved in NorA expression. Menadione was able to inhibit the efflux pump and a genetic expression inhibition of the NorA pump gene was observed. Menadione demonstrated be an efflux pump inhibitor with dual mechanism: affecting the efflux pump directly and indirectly, inhibit the *norA* gene expression. Therefore, new in vivo studies should confirm its viability as an efflux pump inhibitor.

## Figures and Tables

**Table 1:** Primers used in this study.

Gene	Primers	Sequence (5′-3′)	Size (bp) *	Reference
*norA*	NorA-Fw	5′-TTCACCAAGCCATCAAAAAG-3′	620	Couto et al. [26]
	NorA-Rv	5′-CTTGCCTTTCTCCAGCAATA-3′		
*16S*	16S-Fw	5′-GTAGGTGGCAAGCGTTATGCC-3′	228	Lucero et al. [27]
	16S-Rv	5′-CGCACATCAGCGTCAG-3′		

* Base pairs.

**Table 2:** Relative expression of the *norA* gene in *S. aureus* strain in presence of tannic acid. ** *p* < 0.01.

	Control	Menadione + Norfloxacin
**TC mean-*16S***	31,907	31,209
* TC mean-*norA*	26,623	27,903
**∆∆TC (Mean + SD)**	1	0.4534 ± 0.3567 **

* *Threshold* cycle.

## References

[1] Morens D.M., Folkers G.K., Fauci A.S. (2004). The challenge of emerging and re-emerging infectious diseases. Nature.

[2] Wertheim H.F., Melles D.C., Vos M.C., van Leeuwen W., van Belkum A., Verbrugh H.A., Nouwen J.L. (2005). The role of nasal carriage in *Staphylococcus aureus* infections. Lancet Infect. Dis..

[3] Skov R.L., Jensen K.S. (2009). Community-associated meticillin-resistant *Staphylococcus aureus* as a cause of hospital-acquired infections. J. Hosp. Infect..

[4] Emmerson A.M. (2003). The quinolones: Decades of development and use. J. Antimicrob. Chemother..

[5] Fair R.J., Tor Y. (2014). Antibiotics and Bacterial Resistance in the 21st Century. Perspect. Medicin. Chem..

[6] Hernando-Amado S., Blanco P., Alcalde-Rico M., Corona F., Reales-Calderón J.A., Sánchez M.B., Martínez J.L. (2016). Multidrug efflux pumps as main players in intrinsic and acquired resistance to antimicrobials. Drug Resist. Updat..

[7] Webber M.A. (2003). The importance of efflux pumps in bacterial antibiotic resistance. J. Antimicrob. Chemother..

[8] Fajardo A., Martínez-Martín N., Mercadillo M., Galán J.C., Ghysels B., Matthijs S., Cornelis P., Wiehlmann L., Tümmler B., Baquero F. (2008). The Neglected Intrinsic Resistome of Bacterial Pathogens. PLoS ONE..

[9] Ng E.Y., Trucksis W.M., Hooper D.C. (1994). Quinolone resistance mediated by *norA*: Physiologic characterization and relationship to *flqB*, a quinolone resistance locus on the *Staphylococcus aureus* chromosome. Antimicrob. Agen. Chemother..

[10] Lee N., Yuen K.-Y., Kumana C.R. (2003). Clinical Role of β-Lactam/ β -Lactamase Inhibitor Combinations. Drugs.

[11] Poole K. (2007). Efflux pumps as antimicrobial resistance mechanisms. Ann. Med..

[12] Schindler B.D., Jacinto P., Kaatz G.W. (2013). Inhibition of drug efflux pumps in *Staphylococcus aureus*: Current status of potentiating existing antibiotics. Future Microbiol..

[13] Truong J.T., Booth S.L. (2011). Emerging Issues in Vitamin K Research. J. Evid. Based. Complementary Altern. Med..

[14] Hirayama K.B., Speridiao P.G., Fagundes-Neto U. (2006). Ácidos graxos poli-insaturados de cadeia longa. Electron. J. Pediatr. Gastroenterol. Nutr. Liver Dis..

[15] Tintino S.R., Oliveira-Tintino C.D.M., Campina F.F., Weslley Limaverde P., Pereira P.S., Siqueira-Junior J.P., Coutinho H.D.M., Quintans-Júnior L.J., da Silva T.G., Leal-Balbino T.C. (2018). Vitamin K enhances the effect of antibiotics inhibiting the efflux pumps of *Staphylococcus aureus* strains. Med. Chem. Res..

[16] Jiang D., Zhao Y., Wang X., Fan J., Heng J., Liu X., Feng W., Kang X., Huang B., Liu J. (2013). Structure of the YajR transporter suggests a transport mechanism based on the conserved motif A. Proc. Natl. Acad. Sci. USA.

[17] Biasini M., Bienert S., Waterhouse A., Arnold K., Studer G., Schmidt T., Kiefer F., Cassarino T.G., Bertoni M., Bordoli L. (2014). SWISS-MODEL: Modelling protein tertiary and quaternary structure using evolutionary information. Nucleic Acids Res..

[18] Arnold K., Bordoli L., Kopp J., Schwede T. (2006). The SWISS-MODEL workspace: A web-based environment for protein structure homology modelling. Bioinformatics.

[19] Stroet M., Caron B., Visscher K.M., Geerke D.P., Malde A.K., Mark A.E. (2018). Automated Topology Builder Version 3.0: Prediction of Solvation Free Enthalpies in Water and Hexane. J. Chem. Theory Comput..

[20] Morris G.M., Huey R., Lindstrom W., Sanner M.F., Belew R.K., Goodsell D.S., Olson A.J. (2009). AutoDock4 and AutoDockTools4: Automated docking with selective receptor flexibility. J. Comput. Chem..

[21] Goodford P.J. (1985). A computational procedure for determining energetically favorable binding sites on biologically important macromolecules. J. Med. Chem..

[22] Sanner M.F. (1999). Python: A programming language for software integration and development. J. Mol. Graph. Model..

[23] Gasteiger J., Marsili M. (1980). Iterative partial equalization of orbital electronegativity—A rapid access to atomic charges. Tetrahedron.

[24] Weiner S.J., Kollman P.A., Nguyen D.T., Case D.A. (1986). An all atom force field for simulations of proteins and nucleic acids. J. Comput. Chem..

[25] Huey R., Morris G.M., Olson A.J., Goodsell D.S. (2007). A semiempirical free energy force field with charge-based desolvation. J. Comput. Chem..

[26] Couto I., Costa S.S., Viveiros M., Martins M., Amaral L. (2008). Efflux-mediated response of *Staphylococcus aureus* exposed to ethidium bromide. J. Antim Chemoth..

[27] Lucero C.M., Vega O.A., Osorio M.M., Tapia J.C., Antonelli M., Stein G.S., Galindo M.A. (2013). The cancer-related transcription factor Runx2 modulates cell proliferation in human osteosarcoma cell lines. J. Cell Physiol..

[28] da Hora G.C.A., Archilha N.L., Lopes J.L.S., Müller D.M., Coutinho K., Itri R., Soares T.A. (2016). Membrane negative curvature induced by a hybrid peptide from pediocin PA-1 and plantaricin 149 as revealed by atomistic molecular dynamics simulations. Soft Matter.

[29] Kukol A. (2009). Lipid Models for United-Atom Molecular Dynamics Simulations of Proteins. J. Chem. Theory Comput..

[30] Schmid N., Eichenberger A.P., Choutko A., Riniker S., Winger M., Mark A.E., van Gunsteren W.F. (2011). Definition and testing of the GROMOS force-field versions 54A7 and 54B7. Eur. Biophys. J..

[31] Oostenbrink C., Soares T.A., van der Vegt N.F.A., van Gunsteren W.F. (2005). Validation of the 53A6 GROMOS force field. Eur. Biophys. J..

[32] Bussi G., Donadio D., Parrinello M. (2007). Canonical sampling through velocity rescaling. J. Chem. Phys..

[33] Berendsen H.J.C., Postma J.P.M., van Gunsteren W.F., DiNola A., Haak J.R. (1984). Molecular dynamics with coupling to an external bath. J. Chem. Phys..

[34] Darden T., York D., Pedersen L. (1993). Particle mesh Ewald: An N ⋅log( N ) method for Ewald sums in large systems. J. Chem. Phys..

[35] Tironi I.G., Sperb R., Smith P.E., van Gunsteren W.F. (1995). A generalized reaction field method for molecular dynamics simulations. J. Chem. Phys..

[36] Abraham M.J., Murtola T., Schulz R., Páll S., Smith J.C., Hess B., Lindahl E. (2015). GROMACS: High performance molecular simulations through multi-level parallelism from laptops to supercomputers. SoftwareX.

[37] Pontes F.J.S., Rusu V.H., Soares T.A., Lins R.D. (2012). The Effect of Temperature, Cations, and Number of Acyl Chains on the Lamellar to Non-Lamellar Transition in Lipid-A Membranes: A Microscopic View. J. Chem. Theory Comput..

[38] Humphrey W., Dalke A., Schulten K. (1996). VMD: Visual molecular dynamics. J. Mol. Graph..

[39] Singh S., Kalia N.P., Joshi P., Kumar A., Sharma P.R., Kumar A., Bharate S.B., Khan I.A., Boeravinone B. (2017). A Novel Dual Inhibitor of NorA Bacterial Efflux Pump of *Staphylococcus aureus* and Human P-Glycoprotein, Reduces the Biofilm Formation and Intracellular Invasion of Bacteria. Front. Microbiol..

[40] Costa L.M., de Macedo E.V., Oliveira F.A.A., Ferreira J.H.L., Gutierrez S.J.C., Peláez W.J., Lima F.C.A., de Siqueira Júnior J.P., Coutinho H.D.M., Kaatz G.W. (2016). Inhibition of the NorA efflux pump of *Staphylococcus aureus* by synthetic riparins. J. Appl. Microbiol..

[41] Bhaskar B.V., Babu T.M.C., Reddy N.V., Rajendra W. (2016). Homology modeling, molecular dynamics, and virtual screening of NorA efflux pump inhibitors of *Staphylococcus aureus*. Drug Des. Devel. Ther..

[42] Davies J., Wright G.D. (1997). Bacterial resistance to aminoglycoside antibiotics. Trends Microbiol..

[43] Heller D.P., Greenstock C.L. (1994). Fluorescence lifetime analysis of DNA intercalated ethidium bromide and quenching by free dye. Biophys. Chem..

[44] Viveiros M., Martins A., Paixão L., Rodrigues L., Martins M., Couto I., Fähnrich E., Kern W.V., Amaral L. (2008). Demonstration of intrinsic efflux activity of *Escherichia coli* K-12 AG100 by an automated ethidium bromide method. Int. J. Antimicrob. Agents..

[45] Bhardwaj K.A., Mohanty P. (2012). Bacterial Efflux Pumps Involved in Multidrug Resistance and their Inhibitors: Rejuvinating the Antimicrobial Chemotherapy. Recent Pat. Antiinfect. Drug Discov..

[46] Truong-Bolduc Q.C., Zhang X., Hooper D.C. (2003). Characterization of NorR protein, a multifunctional regulator of *norA* expression in *Staphylococcus aureus*. J. Bacteriol..

[47] Dubbs M.D., Gupta R.B. (1998). Solubility of Vitamin E (α-Tocopherol) and Vitamin K 3 (Menadione) in Ethanol−Water Mixture. J. Chem. Eng. Data.

[48] Fournier B., Hooper D.C. (2000). A New Two-Component Regulatory System Involved in Adhesion, Autolysis, and Extracellular Proteolytic Activity of *Staphylococcus aureus*. J. Bacteriol..

[49] Shen L.L., Kohlbrenner W.E., Weigl D., Baranowski J. (1989). Mechanism of quinolone inhibition of DNA gyrase. Appearance of unique norfloxacin binding sites in enzyme-DNA complexes. J. Biol. Chem..

[50] Andrade J.C., Morais Braga M.F.B., Guedes G.M.M., Tintino S.R., Freitas M.A., Quintans L.J., Menezes I.R.A., Coutinho H.D.M. (2017). Menadione (vitamin K) enhances the antibiotic activity of drugs by cell membrane permeabilization mechanism. Saudi J. Biol. Sci..

[51] Chiou T.-J., Zhang J., Ferrans V.J., Tzeng W.-F. (1997). Cardiac and renal toxicity of menadione in rat. Toxicology.

[52] Sikkema J., De Bont J.A.M., Poolman B. (1994). Interactions of cyclic hydrocarbons with biological membranes. J. Biol. Chem..

[53] Turina A.d.V., Nolan M.V., Zygadlo J.A., Perillo M.A. (2006). Natural terpenes: Self-assembly and membrane partitioning. Biophys. Chem..

[54] Lamson D.W., Plaza S.M. (2003). The anticancer effects of vitamin K. Altern. Med. Rev..

[55] Piddock L.J.V. (2006). Multidrug-resistance efflux pumps —Not just for resistance. Nat. Rev. Microbiol..

